# The Effect of Short-Term Aspirin Administration during Programmed Frozen-Thawed Embryo Transfer on Pregnancy Outcomes and Complications

**DOI:** 10.3390/jcm12031064

**Published:** 2023-01-30

**Authors:** Hongcheng He, Dan Qi, Mei Fang, Yizheng Tian, Lei Yan, Jinlong Ma, Yanbo Du

**Affiliations:** 1 Center for Reproductive Medicine, Shandong University, Jinan 250012, China; 2 Key Laboratory of Reproductive Endocrinology of Ministry of Education, Shandong University, Jinan 250012, China; 3 Shandong Provincial Hospital, Shandong First Medical University, Jinan 250021, China

**Keywords:** frozen-embryo transfer, aspirin, pregnancy outcomes, live birth rate, pregnancy complications

## Abstract

Low-dose aspirin is widely used during frozen-embryo transfer (FET) cycles. Its anti-platelet property makes it a potentially useful drug for the prevention of hypertension disorders of pregnancy (HDP). However, the existing evidence about the effect of short-term aspirin administration on pregnancy outcomes is not clear. In our study, we retrospectively investigated women who had their first or second FET cycles at the Reproductive Hospital Affiliated with Shandong University from April 2017 to December 2020. A total of 4454 programmed FET cycles were recruited. According to whether aspirin was administrated in the protocols, the patients were divided into two groups: The Control group (n = 2793, 85 of them using donor sperm) and the Aspirin group (n = 1661, 35 of them using donor sperm). We analyzed the pregnancy outcomes and pregnancy complications of these cycles and observed similar live birth rates. We found that the short-term use of aspirin at a dosage of 50 mg per day for women undergoing programmed FET did not elevate the live birth rate or decrease the incidence of a series of pregnancy complications, including HDP. Based on our experience, short-term administration of low-dose aspirin may not improve the outcomes of young women undergoing frozen-thawed embryo transfer cycles.

## 1. Introduction

For the higher embryo survival rates after the implementation of vitrification in the laboratory [1], elective embryo transfer (ET) policies, and the prevention of ovarian hyperstimulation syndrome (OHSS), the number of frozen-thawed embryo transfer (FET) procedures has shown an increasing prevalence worldwide in the last decade [2]. Some researchers suggested that women would benefit from the FET protocol to reduce the risk of OHSS, low birth weight, placenta previa, placental abruption and perinatal mortality [3,4,5]. The FET procedure includes three available endometrial preparation protocols, namely the natural cycle, stimulation cycle, and program cycle. In clinical practice, the choice of the FET procedure depends on the patient’s situation and the physician’s discretion. In the programmed cycle, spontaneous ovulation is inhibited, and exogenous estrogen and progesterone are provided for the preparation of endometrium [6]. The opportunity for frozen embryo transfer is highly relevant to the thickness of the endometrium and sex hormone levels. A programmed cycle is suitable for patients who suffer from oligo-ovulation or anovulation. Moreover, given the flexibility of schedule for the timing of embryo transfer and the convenience for patients to reduce the frequency of monitoring, the programmed cycle is widely used. Our previous research has discovered that women who have singleton deliveries following FET are more likely to develop preeclampsia and postpartum hemorrhage (PPH) in programming cycles than in natural cycles [7]. The etiology of preeclampsia has not been well explained, and some academics suggested that the mechanism of preeclampsia may be a lack of corpus luteum due to the suppression of ovulation. The major function of the corpus luteum is maintaining normal pregnancy through progesterone secretion. In addition, corpus luteum secrete some important vasoactive substances such as relaxin, angiogenic metabolites of estrogen and vascular endothelial growth factor (VEGF); although not fully confirmed, the absence of these vasoactive substances may have a strong connection with preeclampsia when patients underwent programmed cycle [8,9].

In addition, aspirin, which belongs to the class of non-steroidal anti-inflammatory drugs, is widely used to treat pain, fever and inflammation. Notably, aspirin’s anti-platelet property makes it a potentially useful drug for the prevention of pregnancy-related hypertension (HDP) [10]. In Zhang’s retrospective study, patients with recurrent pregnancy loss (RPL) had poor endometrial blood perfusion, and 50 mg of aspirin per day improved the blood flow of endometrium in patients [11]. In Hsieh’s clinical trial, the administration of aspirin at a dosage of 100 mg per day improved the pregnancy rate in patients with a thin endometrium (≤8 mm) [12]. A meta-analysis also affirmed the effect of aspirin in endometrium preparation during the IVF process; in the research, patients with aspirin administration had a significantly higher live birth rate [13]. However, despite many scholars thinking that low-dose (no more than 100 mg daily) aspirin is relatively safe during pregnancy, a cohort study reveals that aspirin may induce subchorionic hematomas in the early-pregnancy stage [14]. Consequently, the existing researches about the use of aspirin on pregnant women are heterogeneous in the condition of participants, and the existing evidence about the effect of aspirin on gestational outcomes is not clear, especially in the programmed FET population.

Our study aims to investigate the effect of short-term use of low-dose aspirin (50 mg once daily, from the last menstrual period before embryo transfer to 7 weeks of pregnancy) in a programmed cycle of FET on pregnancy outcomes, pregnancy complications, and bleeding risk, and to determine whether low-dose aspirin should be recommended to patients in the programmed cycle.

## 2. Materials and Methods

### 2.1. Study Design

Ours is a retrospective study. Women who had their first or second FET at the Reproductive Hospital Affiliated with Shandong University from April 2017 to December 2020 were recruited. The exclusion criteria were as follows: (i) a history of recurrent pregnancy loss(defined as two or more spontaneous pregnancy losses) or repeated implantation failure (RIF; defined as achieving no successful pregnancy after more than 3 embryo transfers or more than four high-quality embryos), (ii) women who had been diagnosed with hypertension, diabetes mellitus, uterine malformation, adenomyosis, hydrosalpinx, anti-phospholipid syndrome or any other disease that requires regular aspirin use, (iii) pre-implantation genetic testing (PGT) or donor oocyte cycles, and (iv) endometrial thickness <0.7 cm. A total of 4454 programmed FET cycles were recruited. According to whether aspirin was administrated in the protocols, the patients were divided into two groups: the control group (n = 2793, 85 of them using donor sperm) and the aspirin group (n = 1661, 35 of them using donor sperm). We calculated the sample size using the PASS15 software, according to the sample size calculation method of a cohort study and referring to the live birth rate of the previous study [15], when P1 = 0.58 and P2 = 0.53, and set: α = 0.05 and β = 0.8. The minimum sample size is 1550, which indicates that our sample size meets the research needs.

### 2.2. Endometrial Preparation

The subjects took oral estradiol valerate (Progynova, Delpharm Lille; Leverkusen, German) on the second or third day of the menstrual cycle at a dose of 4–8 mg, lasting for 10–14 days to promote endometrial proliferation. The dosage and duration of estradiol were raised until the endometrial thickness reached a proper state for embryo transfer (commonly at least 0.7 cm), at which time vaginal progestin (200 mg once daily, Utrogestan, Besins Healthcare; Chatswood, NSW, Australia) and oral dydrogesterone (20 mg twice daily, Duphaston, Abbott; Chicago, IL, USA) were added. The patients in the aspirin group received aspirin 50 mg per day orally after the last menses. Physicians would consider the patients’ maternity history, gynecological ultrasound, and sex hormone levels to decide whether to give aspirin treatment. If conception was confirmed, the subjects had exogenous estrogen for 7 weeks and exogenous progesterone for 10 weeks. In the Aspirin group, aspirin would be withdrawn after the embryocardia beats showed up in 7 weeks. We defined this usage of aspirin as short-term usage.

### 2.3. Outcomes

The primary outcome of our study was the live birth rate, which was defined as the delivery of at least one viable neonate after 28 gestational weeks. Clinical pregnancy was defined as the detection of an intrauterine gestational sac by transvaginal ultrasonography after 6 weeks of FET. Biochemical pregnancy was defined as a blood β-hCG level of 10 IU/L two weeks after embryo transfer. Preterm birth was defined as infants born after fewer than 37 weeks of pregnancy. Post-term birth was defined as birth after a pregnancy duration of 42 weeks. Miscarriage was defined as the loss of pregnancy less than 24 weeks gestation. Ectopic pregnancy was defined as the presence of an extra-uterine gestational sac. 

In addition, the complications of pregnancy were also analyzed. The outcome included the following five indicators of pregnancy complications: HDP was defined as diastolic blood pressure ≥90 mmHg or systolic blood pressure ≥140 mmHg at least twice after the 20th gestational week associated with or without proteinuria and fluid retention. Gestational diabetes mellitus (GDM) was defined as any degree of glucose intolerance with onset or first recognition during pregnancy, PPH was defined as blood loss of >500 mL after vaginal delivery or >1000 mL after cesarean delivery, placental complications include placenta previa, placental abruption, heteromorphic placenta, and placenta accrete, the subchorionic hematoma was defined as sonolucent fluid collections between the chorion and the uterine wall.

### 2.4. Statistical Analysis

Continuous variables are presented as the mean and standard deviation. Differences in characteristics between groups were analyzed using a Student’s *t*-test. Categorical variables are presented as percentages and compared by the chi-square test between groups. Logistic regression was applied for multivariate analysis of pregnancy outcomes and pregnancy complications, confounding factors such as maternal age, BMI (body mass index), endometrial thickness, and AMH (anti-Mullerian hormone) based on the data. All data were processed by IBM SPSS Statistics 26.0. A two-sided *p*-value less than 0.05 was considered statistically significant.

### 2.5. Ethics Statement

The study was approved by the Institutional Review Board of the Reproductive Hospital Affiliated with Shandong University, and the process of data extraction was done in compliance with data protection rules.

## 3. Result

### 3.1. Characteristics of Study Population

A total of 4454 programmed FET cycles were collected in this retrospective study. 1661 (37.29%) patients were treated with aspirin in their FET process, while 2793 (62.71%) patients received no treatment with aspirin.

The baseline characteristics of the patients in the two groups are shown in Table 1. Compared with the control group, the maternal age, blood glucose, and baseline FSH (follicle-stimulating hormone) were higher in the Aspirin group, and the endometrial thickness, baseline AMH level, and polycystic ovary syndrome (PCOS) ratio were lower in the Aspirin group. These differences were statistically significant. While there were no statistically significant differences between the two groups in terms of systolic pressure, diastolic pressure, baseline luteinizing hormone (LH), baseline estradiol, the number of embryos transferred, or the duration of the attempt to conceive (*p* > 0.05).

### 3.2. The Relationships between Aspirin and Pregnancy Outcomes and Complications

The pregnancy outcomes in the two groups are shown in Table 2. We observed similar live birth rates (56.11% vs. 59.36%), clinical pregnancy rates (66.23% vs. 68.67%), preterm birth rates (7.16% vs. 6.52%), post-term birth rates (0.36% vs. 0.07%), miscarriage rates (9.75% vs. 9.17%) and ectopic pregnancy rates (0.48% vs. 0.25%) between two groups. The association of aspirin with these pregnancy outcomes was not statistically significant after adjusting for covariates.

Furthermore, the relationships between aspirin and pregnancy complications are shown in Table 3. Only when women had achieved clinical pregnancies or delivered one or more live infants were included in the analysis. There were also no significant differences in terms of HDP (9.00% vs. 6.78%), GDM (9.00% vs. 8.65%), PPH (1.07% vs. 0.66%), placental previa (1.36% vs. 0.89%), and subchorionic hematoma (17.60% vs. 18.15%) between the two groups after adjusting for baseline characteristics in the multivariable logistic regression model.

### 3.3. Subgroup Analysis

For further analysis of the effect of aspirin on pregnancy outcomes and pregnancy complications in PCOS and obesity patients, a subgroup analysis was performed. Supplemental Table S1 shows the baseline characteristics of women in subgroups. The endometrial thickness was significantly lower in the Aspirin group compared with the control group in all subgroups. We found similar pregnancy outcomes and pregnancy complication rates between the aspirin and control groups, whether PCOS or non-PCOS patients and whether obese or normal-weight patients (Table 4 and Table 5).

## 4. Discussions

In our study, women undergoing a programmed FET cycle did not benefit from low-dose aspirin in pregnancy outcomes, including the live birth rate, clinical pregnancy rate, biochemical pregnancy rate, preterm birth rate, post-term birth rate, miscarriage rate, and ectopic pregnancy rate, which was consistent with Robab et al. [13], He reported that the application of 80 mg of oral aspirin had no positive effect on the pregnancy outcomes, including the clinical pregnancy rate, the chemical pregnancy rate and the abortion rate of women who underwent IVF. It is noteworthy that in Robab’s research, the endometrial thickness in the aspirin group was significantly lower than in the control group, which is consistent with our findings. Thin endometrium has been thought to be associated with poor pregnancy outcomes, and the bias may affect the results [16]. The pregnancy outcomes of our study were consistent with the research from Haapsamo et al., in their randomized clinical trial, they transferred fresh embryos to 456 women, and 227 of them received 100 mg of aspirin per day. From the research, they demonstrated that low-dose aspirin decreased the incidence of non-optimal uterine hemodynamics, but there was no significant difference in uterine artery vascular impedance on the day of embryo transfer, and the results showed similar live birth rates and the incidence of hypertensive pregnancy complications between aspirin group and control group [17]. Low-dose aspirin was reported to improve the uterine artery pulsatility index, but there was no evidence to certify the correlation between the uterine artery doppler index and pregnancy outcomes [18,19,20]. A randomized control study conducted by Tahereh and his colleagues has shown inconsistent results, indicating that low-dose aspirin administration resulted in a higher implantation rate, clinical pregnancy rate and live birth rate in women undergoing the FET cycle. But the number of participants in this study was only 60, and further randomized clinical studies in larger populations are needed to confirm this result [12].

Our study observed that aspirin administration has no effect on the risk of pregnancy complications. Firstly, there was no significant difference in the risk of HDP between the Aspirin group and the Control group, which was consistent with Haapsamo’s [17]. The USPSTF (US Preventive Services Working Group) suggested the use of aspirin at a dosage of 81 mg per day for preeclampsia prevention, but only for women who were at high risk of preeclampsia [21]. SMFM (Society for Maternal-Fetal Medicine) also did not recommend low-dose aspirin for routine use when IVF is the only indication for preeclampsia [22]. Stephanie et al., in their meta-analysis of the results of 16 randomized controlled trials (18,907 participants), found that aspirin did reduce the risk of preterm preeclampsia, but not term preeclampsia, and only when it was initiated at ≤16 weeks of gestation and at a daily dose of ≥100 mg [23]. Similar to the Research from Daniel et al., they found that women who have a higher risk of preterm preeclampsia benefited from 150 mg aspirin per day during pregnancy, reducing the incidence of preterm preeclampsia [24]. A pilot study conducted by Marieke et al. established that women with 100 mg aspirin administration during IVF treatment and the first trimester had a decreased incidence of hypertension complications. Patients in their study had a history of implantation failure, which may be a potential risk factor for preeclampsia [25]. In our study, we excluded patients who had a history of RPL or RIF, which were thought to be risk factors for HDP. A systematic review of large cohort studies involving over 1000 women revealed that antiphospholipid antibody syndrome, prior pre-eclampsia, chronic hypertension, pre-gestational diabetes, and BMI > 30 were all potential risk factors for pre-eclampsia, and the application of assisted reproductive technology was also included [26]. In conclusion, only when aspirin reaches a certain dosage which may be ≥100 mg [27], could it have an effect on the prevention of HDP. And people at higher risk of preeclampsia may likely benefit from low-dose aspirin during pregnancy, and the effect is linked to the dose of aspirin. Therefore, the association between aspirin and HDP in the FET program population needs further study.

Moreover, no significant differences in pregnancy rate and pregnancy complications were observed in subgroup analysis when we subdivided patients into PCOS/non-PCOS and obesity/non-obesity. Patients with PCOS or obesity were considered to have a higher risk of HDP and preeclampsia [27,28]. Thromboxane A2 (TXA2) has a strong connection with the pathogenesis of preeclampsia, and a study concluded that 60 mg of aspirin per day could decrease the Thromboxane B2 (an indirect measure of TXA2) level in women, whether overweight or not, and they suggested a higher dose of aspirin for overweight patients [29]. A study from Namavar revealed that 100 mg of aspirin per day in patients with PCOS could neither increase the clinical pregnancy rate nor decrease the OHSS rate [30]. It seems that 50 mg of aspirin daily could not reduce the rate of HDP and preeclampsia in either PCOS or obese patients. There is limited research on aspirin in PCOS, and aspirin administration in these two groups needs more investigation, especially to find out the optimal dose in different populations.

Furthermore, there’s no statistically significant difference in the outcome of placenta complications between the two groups. The placenta, which is the unique exchange organ between mother and fetus, undertakes the exchange of nutrients and waste and secretes a number of hormones that play an important role in maternal metabolism [31]. Abnormal placentation is strongly associated with distinct pregnancy complications such as miscarriage, preterm birth, and preeclampsia [32]. In our study, patients in the aspirin group took aspirin orally until 7 weeks of pregnancy, while the placenta gradually replaced the yolk sac at about 10 weeks of pregnancy. Insufficient duration of aspirin administration may be the reason for this result.

Next, our study showed that 50 mg per day of aspirin administration in a programmed FET cycle would not increase the risk of PPH and subchorionic hemorrhage. A large cohort study revealed that more than 150 mg per day of aspirin treatment would increase postpartum bleeding and PPH [33]. A meta-analysis consisting of 13 trials revealed that the use of aspirin in people without cardiovascular diseases had an increased risk of major bleeding and suggested that we should be more careful when prescribing aspirin for cardiovascular event prevention [34]. Collectively, high-dose aspirin may be superior to relatively low-dose aspirin in the effect of HDP prevention, but it also has a higher risk of bleeding. A large randomized controlled trial is required to explore the optimal dose of aspirin when used in pregnant women for HDP prophylaxis. In our study, we also reported a similar incidence of placental complications. It’s widely accepted that reduced placental perfusion is strongly associated with preeclampsia. The effect of aspirin on placental perfusion maybe need more research.

Notably, recent literature reported that, in nulliparous women, the incidence of preeclampsia might increase because of lower overall sperm exposure [35], which means that donor sperm embryo transfer and husband embryo transfer may affect the result of pregnancy complications. In our study, donor sperm IVF/ICSI accounted for less than 5% of total cycles, and there’s no statistical significance in donor sperm cases between the two groups. Future studies should take note of this and focus on autologous cycles when investigating pregnancy complications.

Our study also showed that aspirin has no relation to the rate of GDM. At present, there are few studies on aspirin and GDM, and no powerful evidence has been established on the association between GDM and aspirin. The effect of aspirin on pregnancy complications in different populations needs further study. 

## 5. Strengths and Limitations

The key strength of our study is the large sample size, which allowed for the subgroup analysis according to the diagnoses of obesity and PCOS. Moreover, we comprehensively analyzed both the pregnancy outcomes and pregnancy complications under the influence of low-dose aspirin. In addition, our study population focused on the FET population, especially those who underwent their first or second FET and assessed the effects of short-term aspirin in women with different conditions (obesity or PCOS). However, all women in our study took the same dose of aspirin (50 mg per day), and we failed to provide evidence for the effect of different doses of aspirin per day on the rate of HDP and subchorionic hematoma. Furthermore, regardless of whether or not clinical pregnancy was achieved, patients in the aspirin group received only 7 weeks of aspirin treatment, a relatively short duration. Despite some existing evidence confirming the effect of aspirin administration in preeclampsia prevention when the medication is initiated before 16 weeks of gestation [36], the appropriate medication timing of aspirin during pregnancy needs further exploration. Besides, there’s no absolute standard for patients who need medication, and the choice of aspirin treatment depends to some extent on physicians’ preferences. A well-designed, multi-center RCT should be conducted to investigate the population suitable for aspirin and the appropriate dosage and duration of aspirin administration.

## 6. Conclusions

In conclusion, our study revealed that the short-term (7-week) use of aspirin at a dosage of 50 mg per day for women undergoing programmed FET did not provide additional benefits. The two groups had similar live birth rates and a series of pregnancy complications. We should be prudent about taking aspirin during FET cycles before embryo transfer. Our study was conducted only in patients without specific risk factors, and a small proportion of donor sperm cycles were included in our study. Future research could focus on the effects of aspirin in specific populations, such as those suffering from recurrent miscarriages or frozen embryo transfers with repeated implantation failures. More randomized controlled trials of high quality are required for further research.

## Figures and Tables

**Table 1:** General population characteristics of study subjects.

	Aspirin Group	Control Group	*p*-Value
	(n = 1661)	(n = 2793)	
Age (years)	30.74 ± 4.64	29.94 ± 4.14	<0.001
BMI (kg/m^2^)	24.64 ± 3.86	24.43 ± 3.76	0.088
Duration of infertility (years)	3.81 ± 2.70	3.89 ± 2.52	0.313
Endometrial thickness in FET (cm)	0.88 ± 0.13	0.93 ± 0.14	<0.001
Blood glucose (mmol/L)	5.29 ± 0.55	5.26 ± 0.49	0.047
Systolic pressure (mmHg)	115.02 ± 12.70	114.91 ± 12.48	0.778
Diastolic pressure (mmHg)	69.29 ± 9.89	69.13 ± 9.60	0.595
Baseline FSH	6.21 ± 2.94	6.02 ± 1.75	0.005
Baseline LH	7.01 ± 5.22	7.30 ± 5.04	0.065
Baseline E2	41.78 ± 36.44	41.71 ± 39.68	0.953
Baseline AMH	6.09 ± 4.41	6.62 ± 4.49	<0.001
**Stage of embryo transferred**			0.035
Day 5	1244 (74.89)	2186 (78.27)	
Day 6	396 (23.84)	575 (20.59)	
Other	21 (1.26)	32 (1.14)	
**Sperm sources**			0.061
Donor sperm	35 (2.11)	85 (3.04)	
Husband sperm	1626 (97.89)	2708 (96.96)	
**Number of embryos transferred**			0.106
1	1586 (95.48)	2694 (96.46)	
2	75 (4.52)	99 (3.54)	
PCOS	717 (43.17)	1335 (47.80)	0.003
Endometriosis	46 (2.77)	102 (3.65)	0.112

Table 1 displays the baseline characteristics of the two groups. PCOS, polycystic ovary syndrome; BMI, body mass index; FSH, follicle-stimulating hormone; LH, luteinizing hormone; AMH, anti-Müllerian hormone.

**Table 2:** Relationships between aspirin and pregnancy outcomes.

	Aspirin Group	Control Group	aOR (95% CI)	*p* Value
	(n = 1661)	(n = 2793)		
Live birth rate	932 (56.11)	1658 (59.36)	1.003 (0.880–1.144)	0.960
Clinical pregnancy rate	1100 (66.23)	1918 (68.67)	1.024 (0.893–1.175)	0.730
Biochemical pregnancy rate	1235 (74.35)	2109 (75.51)	1.086 (0.937–1.259)	0.275
Preterm birth rate	119 (7.16)	182 (6.52)	1.141 (0.894–1.458)	0.290
Postterm birth rate	6 (0.36)	2 (0.07)	4.854 (0.954–24.713)	0.057
Miscarriage rate	162 (9.75)	256 (9.17)	1.026 (0.830–1.267)	0.813
Ectopic pregnancy rate	8 (0.48)	7 (0.25)	1.840 (0.646–5.243)	0.253

The pregnancy outcomes in the two groups are shown in Table 2. Pregnancy outcomes were compared with logistic regression analysis, and the following confounding effects were controlled for, including endometrial thickness, age, BMI, blood glucose, baseline FSH, baseline AMH, the timing of embryo transfer, and PCOS.

**Table 3:** Relationships between Aspirin and pregnancy complications.

	Aspirin Group	Control Group	aOR (95% CI)	*p*-Value
	No./Total No. (%)			
HDP *	99/1100 (9.00)	130/1918 (6.78)	1.306 (0.985–1.732)	0.064
GDM *	99/1100 (9.00)	166/1918 (8.65)	0.991 (0.759–1.295)	0.950
Placental complications *	15/1100 (1.36)	17/1918 (0.89)	1.673 (0.822–3.405)	0.156
PPH #	10/932 (1.07)	11/1658 (0.66)	1.783 (0.743–4.281)	0. 195
Subchorionic hematoma #	164/932 (17.60)	301/1658 (18.15)	0.950 (0.768–1.176)	0.638

Note: HDP, hypertension disorders of pregnancy; GDM, gestational diabetes mellitus; PPH, postpartum hemorrhage. Pregnancy complications were compared with logistic regression analysis, and the following confounding effects were controlled for: including endometrial thickness, age, BMI, and baseline FSH. When we analyze HDP, the systolic and diastolic pressures are adjusted; when we analyze GDM, the blood glucose is adjusted. * Evaluation was performed in all clinical pregnancies. # Evaluation was performed in all live births.

**Table 4:** Relationships between aspirin and pregnancy outcomes in different subgroups.

	BMI < 28 kg/m^2^				BMI ≥ 28 kg/m^2^			
	Aspirin Group	Control Group	aOR (95% CI)	*p*-Value	Aspirin Group	Control Group	aOR (95% CI)	*p*-Value
	(n = 1340)	(n = 2309)			(n = 321)	(n = 484)		
Live birth rate	772 (57.61)	1387 (60.07)	1.030 (0.893–1.188)	0.687	160 (49.84)	271 (55.99)	0.818 (0.613–1.090)	0.171
Clinical pregnancy rate	897 (66.94)	1583 (68.56)	1.061 (0.913–1.233)	0.439	203 (63.24)	335 (69.21)	0.798 (0.589–1.080)	0.143
Biochemical pregnancy rate	998 (74.48)	1742 (75.44)	1.094 (0.930–1.287)	0.277	237 (73.83)	367 (75.83)	0.942 (0.678–1.309)	0.721
Preterm birth rate	88 (6.57)	143 (6.19)	1.123 (0.847–1.488)	0.420	31 (9.66)	39 (8.06)	1.219 (0.741–2.006)	0.435
Postterm birth rate	4 (0.30)	2 (0.09)	3.943 (0.701–22.164)	0.119	2 (0.62)	0	NA	NA
Miscarriage rate	121 (9.03)	192 (8.32)	1.058 (0.828–1.350)	0.653	41 (12.77)	64 (13.22)	0.945 (0.619–1.443)	0.794
Ectopic pregnancy rate	7 (0.52)	7 (0.30)	1.554 (0.530–4.559)	0.422	1 (0.31)	0	NA	NA
	**PCOS**				**Non-PCOS**			
	**Aspirin Group**	**Control Group**	**aOR (95% CI)**	***p*-Value**	**Aspirin Group**	**Control Group**	**aOR (95% CI)**	***p*-Value**
	**(n = 717)**	**(n = 1335)**			**(n = 944)**	**(n = 1458)**		
Live birth rate	436 (60.81)	822 (61.57)	1.019 (0.842–1.233)	0.846	496 (52.54)	836 (58.34)	0.965 (0.812–1.148)	0.690
Clinical pregnancy rate	519 (72.38)	955 (71.54)	1.090 (0.887–1.340)	0.412	581 (61.55)	963 (66.05)	0.951 (0.796–1.137)	0.583
Biochemical pregnancy rate	576 (80.33)	1049 (78.58)	1.150 (0.914–1.447)	0.233	659 (69.81)	1060 (72.70)	1.014 (0.839–1.225)	0.885
Preterm birth rate	67 (9.34)	93 (6.97)	1.384 (0.991–1.931)	0.056	52 (5.51)	89 (61.04)	0.884 (0.615–1.270)	0.504
Postterm birth rate	1 (0.14)	0	NA	NA	5 (0.53)	2 (0.14)	4.036 (0.764–21.310)	0.100
Miscarriage rate	81 (11.30)	132 (9.89)	1.123 (0.833–1.514)	0.447	81 (8.58)	124 (8.50)	0.939 (0.696–1.267)	0.682
Ectopic pregnancy rate	3 (0.42)	2 (0.15)	3.058 (0.491–19.063)	0.231	5 (0.53)	5 (0.34)	1.392 (0.388–4.991)	0.611

Note: NA, not available, which means missing value.

**Table 5:** Relationships between aspirin and pregnancy complications in different subgroups.

	BMI < 28 kg/m^2^				BMI ≥ 28 kg/m^2^			
	Aspirin Group	Control Group	aOR (95% CI)	*p*-Value	Aspirin Group	Control Group	aOR (95% CI)	*p*-Value
	No./Total No. (%)				No./Total No. (%)			
HDP *	70/897 (7.80)	93/1583 (5.87)	1.328 (0.953–1.851)	0.094	29/203 (14.29)	37/335 (11.04)	1.264 (0.735–2.173)	0.397
GDM *	73/897 (8.14)	119/1583 (7.52)	1.032 (0.756–1.409)	0.842	26/203 (12.81)	47/335 (14.03)	0.863 (0.513–1.452)	0.578
Placental complications *	13/897 (1.45)	13/1583 (0.82)	1.931 (0.877–4.253)	0.103	2/203 (0.99)	4/335 (1.19)	0.917 (0.164–5.135)	0.921
PPH #	10/772 (1.30)	9/1387 (0.65)	2.250 (0.892–5.676)	0.086	0	2/271 (0.74)	NA	NA
Subchorionic hematoma #	134/772 (17.36)	259/1387 (18.67)	0.893 (0.706–1.129)	0.344	30/160 (18.75)	42/271 (15.50)	1.256 (0.747–2.112)	0.391
	**PCOS**				**Non-PCOS**			
	**Aspirin Group**	**Control Group**	**aOR (95% CI)**	***p-*** **Value**	**Aspirin Group**	**Control Group**	**aOR (95% CI)**	***p-*** **Value**
	**No./Total No. (%)**				**No./Total No. (%)**			
HDP *	44/519 (10.09)	63/955 (7.66)	1.249 (0.824–1.891)	0.249	55/581 (11.09)	67/963 (8.01)	1.362 (0.925–2.006)	0.118
GDM *	63/519 (14.45)	95/955 (11.56)	1.127 (0.794–1.597)	0.504	36/581 (7.06)	71/581 (8.49)	0.809 (0.530–1.234)	0.325
Placental complications *	5/519 (1.15)	7/955 (0.85)	1.426 (0.441–4.611)	0.554	10/581 (2.02)	10/581 (1.20)	1.830 (0.747–4.483)	0.186
PPH #	4/436 (0.92)	5/823 (0.61)	1.407 (0.364–5.431)	0.620	6/496 (1.21)	6/836 (0.72)	1.905 (0.598–7.065)	0.275
Subchorionic hematoma #	74/436 (16.97)	147/823 (17.88)	0.937 (0.686–1.281)	0.685	90/496 (18.15)	154/836 (18.42)	0.960 (0.717–1.285)	0.783

* Evaluation was performed in all clinical pregnancies. # Evaluation was performed in all live births.

## Data Availability

The raw data used in this study are available from the corresponding author upon reasonable request.

## Supplementary Materials

The following supporting information can be downloaded at: https://www.mdpi.com/article/10.3390/jcm12031064/s1, Table S1: Differences in general population characteristics of obesity in ASA group and control group.
