# Supplementary material for: The Effect of Short-Term Aspirin Administration during Programmed Frozen-Thawed Embryo Transfer on Pregnancy Outcomes and Complications
Source: J Clin Med. 2023 Jan 30;12(3):1064. doi: 10.3390/jcm12031064 (PMC9918171; doi:10.3390/jcm12031064)
Supplement: Supplementary file 1 [file jcm-12-01064-s001.zip › jcm-2082577-supplementary.pdf]

Supplemental Table S1.Differences in general population characteristics of obesity in ASA group and control group

	**BMI<28kg/m²**			**BMI≥28kg/m²**		
	**Aspirin group (n=1340)**	**Control group (n=2309)**	***P* value**	**Aspirin group (n=321)**	**Control group (n=484)**	***P* value**
Age(years)	30.77±4.64	29.87±4.07	<0.001	30.63±4.67	30.29±4.45	0.301
BMI(kg/m²)	23.25±2.72	23.18±2.66	0.465	30.44±2.07	30.43±2.11	0.950
Duration of infertility(years)	3.66±2.63	3.75±2.45	0.323	4.43±2.88	4.58±2.72	0.459
Endometrial thickness in FET(cm)	0.87±0.12	0.92±0.13	<0.001	0.93±0.15	0.96±0.15	0.006
Blood glucose(mmol/L)	5.25±0.45	5,23±0.44	0.204	5.46±0.82	5.40±0.66	0.215
Systolic pressure(mmHg)	113.57±12.28	113.56±12.07	0.986	121.10±12.65	121.37±12.42	0.764
Diastolic pressure(mmHg)	68.21±9.48	68.11±9.17	0.754	73.78±10.30	73.98±10.11	0.790
Baseline FSH	6.34±3.14	6.09±1.80	0.002	5.68±1.77	5.69±1.50	0.925
Baseline LH	7.14±5.30	7.44±5.19	0.099	6.46±4.83	6.66±4.21	0.527
Baseline E2	42.37±38.54	42.14±40.80	0.872	39.33±25.79	39.62±33.78	0.895
Baseline AMH	6.08±4.46	6.70±4.53	<0.001	6.11±4.23	6.24±4.32	0.670
Stage of embryo transferred			0.053			0.156
Day 5	1006(75.07)	1810(78.39)		238(74.14)	376(77.69)	
Day 6	318(23.73)	469(20.31)		78(24.30)	106(21.90)	
Other	16(1.19)	30(1.30)		5(1.56)	2(0.41)	
Number of embryos transferred			0.074			0.989
1	1277(95.30)	2228(96.49)		309(96.26)	466(96.28)	
2	63(4.70)	81(3.51)		12(3.74)	18(3.72)	
PCOS	532(39.70)	1057(45.78)	<0.001	185(57.63)	278(57.44)	0.956
Endometriosis	42(3.13)	94(4.07)	0.150	4(1.25)	8(1.65)	0.641

	PCOS			non-PCOS		
	**Aspirin group (n=717)**	**Control group (n=1335)**	***P* value**	**Aspirin group (n=944)**	**Control group (n=1458)**	***P* value**
Age(years)	29.28±3.75	29.01±3.58	0.105	31.85±4.94	30.80±4.42	<0.001
BMI(kg/m^2)	25.24±4.10	24.89±3.90	0.057	24.17±3.60	24.01±3.59	0.286
Duration of infertility(years)	3.95±2.49	4.06±2.48	0.340	3.70±2.84	3.74±2.55	0.757
Endometrial thickness in FET(cm)	0.89±0.13	0.93±0.13	<0.001	0.88±0.13	0.92±0.14	<0.001
Blood glucose(mmol/L)	5.28±0.49	5.25±0.44	0.241	5.30±0.59	5.27±0.53	0.131
Systolic pressure(mmHg)	115.53±12.30	115.40±12.62	0.821	114.64±12.99	114.47±12.34	0.750
Diastolic pressure(mmHg)	70.22±9.49	69.83±9.63	0.380	68.58±10.12	68.49±9.52	0.818
Baseline FSH	5.60±1.54	5.60±1.44	0.983	6.68±3.59	6.40±1.92	0.013
Baseline LH	8.99±6.37	9.19±5.71	0.473	5.51±3.45	5.58±3.56	0.608
Baseline E2	44.89±38.74	44.30±46.47	0.773	39.41±34.42	39.33±32.05	0.951
Baseline AMH	9.01±4.43	9.25±4.39	0.250	3.87±2.81	4.22±2.98	0.004
Stage of embryo transferred			0.391			0.243
Day 5	593(82.71)	1134(84.94)		651(68.96)	1052(72.15)	
Day 6	120(16.74)	193(14.46)		276(29.24)	382(26.20)	
Other	4(0.55)	8(0.60)		17(1.80)	24(1.65)	
Number of embryos transferred			0.590			0.049
1	681(94.98)	1275(95.51)		905(95.87)	1419(97.33)	
2	36(5.02)	60(4.49)		39(4.13)	39(2.67)	
Endometriosis	12(1.67)	35(2.62)	0.171	34(3.60)	67(4.60)	0.236

PCOS, polycystic ovary syndrome; BMI, body mass index; FSH, follicle-stimulating hormone; LH, luteinizing hormone; AMH, anti-Müllerian hormone;
